# *Herpes Simplex Virus* Meningitis in Children in South East of Caspian Sea, Iran

**DOI:** 10.5812/jjm.8599

**Published:** 2014-01-01

**Authors:** Somayeh Azadfar, Fatemeh Cheraghali, Abdolvahab Moradi, Naeme Javid, Alijan Tabarraei

**Affiliations:** 1 Department of Microbiology, Infectious Diseases Research Centre, Golestan University of Medical Sciences, Gorgan, IR Iran

**Keywords:** Herpes Simplex Virus, Meningitis, CSF, PCR, Children

## Abstract

**Background:**

*Herpes simplex virus* (HSV) is a member of *Herpesviridae *and a leading cause of human viral diseases. Meningitis occurs as a complication of HSV-1 or HSV-2 primary infection.

**Objectives:**

We aimed to evaluate HSV meningitis in children in Gorgan province, Iran.

**Patients and Methods:**

Forty-five cerebrospinal fluid samples were taken from children referred with meningitis symptoms. Samples with negative bacterial culture results were tested for viral, biochemical and cytological assays. DNA extraction and PCR were performed.

**Results:**

HSV-1 detected in 4 (8.8%) samples without any HSV-2 infections. Cases with positive results had fever and CSF pleocytosis. Vomiting, headache and higher count of WBC were observed in 3, 2 and 3 cases respectively. The cerebrospinal fluid (CSF) glucose and protein levels were normal and 3 cases showed positive C-reactive protein (CRP) results. Also erythrocyte sedimentation rate (ESR) was higher than normal in all positive cases.

**Conclusions:**

Distribution of HSV types in children with meningitis in our area predominantly was type 1 compared with type 2, which has been reported more in other area.

## 1. Background

Meningitis is a widespread central nervous system diseases especially in children and infants (1). Viruses are the most common cause of aseptic meningitis in children and the study on etiology, clinical manifestations and diagnosis of viral meningitis has priority. Viral meningitis is generally similar to bacterial meningitis with less severity and differential diagnosis. In children, clinical manifestations of viral meningitis vary according to their age, immune status, and etiologic agents (2). 

Neonates are more at the increased risk of severe systemic diseases, particularly nervous system involvement with *Herpes simplex virus* (HSV) (3). It has been reported that the herpes virus family collectively involved about 4% of viral meningitis cases which more are cases caused by HSV-2. As HSV-2 is restricted to aseptic meningitis in CNS infections, HSV-1 is the most common cause of herpetic encephalitis; however, some recent studies showed the diversion of common herpes virus meningitis causes from HSV-2 to HSV-1. Viral meningitis can occur at any age but is mostly common among young children. Currently, HSV is the second causes of viral meningitis in adolescents and adults living in developing countries (4, 5). Initial laboratory evaluations of a child with suspected meningitis included blood tests and lumbar puncture. The initial blood and CSF tests are essential for and should be bacterial cultures or CBC with differential and platelet count should be performed. Lumbar puncture also may provide relief symptom in patients with viral meningitis (6, 7).

 The diagnosis of viral meningitis is based on culture results, direct fluorescent antibody (DFA), skin biopsy and molecular methods for detection of viral DNA. PCR is a method of choice for rapid, sensitive, and specific identification of HSV (8). Regarding to the importance of application of new techniques for viral meningitis detection for understanding of feature of epidemiology, pathogenesis, management, prognosis, therapy and prevention of viral meningitis, molecular diagnosis are pioneer. 

## 2. Objectives 

The aim of this study was to show the distribution of aseptic meningitis caused by herpes simplex viruses, using PCR in children with respect to clinical manifestations and cerebrospinal fluid (CSF) laboratory findings. As far as we know, this is the first report of molecular detection of viruses in CSF from Gorgan, south east of Caspian sea, Iran.

## 3. Patients and Methods 

### 3.1. Patients

In this study CSF samples were taken from children referred to Taleghani hospital with meningitis symptoms from June 2008 to September 2010. Demographic, clinical, biochemical and cytological data were collected. Children were clinically examined by the specialist and then from suspicious cases, samples were collected by lumbar puncture of spinal cord. Three separated tubes of CSF (3-5 mL) were transported to the laboratory for bacterial culture, biochemical analyses, cell count and molecular recognition. Blood and urine samples were taken for microscopic, biochemical assays, cell count and bacterial growth. PCR was performed on samples collected from patients with CSF pleocytosis and negative bacterial culture. Clinically, most of children had fever, vomiting, headache and fontanel.

### 3.2. Extraction

For DNA extraction, high pure viral nucleic acid kit (Roche, USA) was used according to manufacturer instruction. DNA was stored at -80°C until PCR performed. 

### 3.3. Polymerase Chain Reaction (PCR)

Glycoprotein D gene sequences were used as primers (Table 1) for PCR (9). 

For the reaction, optimized PCR amplifications of HSV-1 and HSV-2 were performed in a solution with a total volume of 50 μL containing 1X PCR buffer, 1.5 mM MgCl_2_, 0.2 mM each of deoxynucleotide triphosphate (dNTPs), 10 pmol of each primers and 2.5 unit Taq DNA polymerase and nuclease free water (Fermentas, USA). 

**Table 1.** Primer Sequences Used for PCR

Virus	Genome Region Amplified	Primer Sequence
**HSV-1**	Glycoprotein D	
		5' CGA AGA CGT CCG GAA ACA AAC 3'
		5' CGG TGC TCC AGG ATA AAA 3'
**HSV-2**	Glycoprotein D	
		5'GGA CGA GGC CCG AAA GCA CA3'
		5' CGG TGC TCC AGG ATA AA 3'

For both HSV-1 and HSV-2, the PCR program was as follow: 1 cycle at 94°C for 40 seconds followed by 33 cycles at 94°C for 20 seconds, 50 °C for 20 seconds and 72°C for 20 seconds.

Final extension was performed at 72°C for 1 minute. PCR product was loaded on 2 % w/v agarose gel and stained with ethidium bromide. The 298 bp product was visualized under UV translumination. Negative and positive controls were used.

### 3.4. Statistical Analysis

 Data entered in SPSS (version 18) and statistical analysis performed with Chi Square test. All cases with P < 0.05 were considered significant.

## 4. Results

Of forty-five patients, 27 cases (60%) were male and 18 cases (40%) female. Children were between 1 month and 10 years old. The mean age of patient was 3.08 years with range of 5 months to 7 years (Table 2). 

**Table 2.** Distribution of HSV-Positive Cases Based on Their Gender, Age and Residency

Cases	No. (%)
**Positive**	
HSV-1	4 (8.8)
HSV-2	0 (0)
**Gender**	
Male	2 (50)
Female	2 (50)
**Age, mo**	
0-24	2 (4/4)
25-48	1 (2.2)
49-72	0 (0)
72-96	1 (2.2)
**Residency**	
Urban	2 (50)
Rural	2 (50)

The same ratio of male to female, was seen in positive cases. Among samples considered for aseptic meningitis, only HSV-1 detected in 4 (8.8%) samples without any positive case for HSV-2. Clinical assay showed a 5-month infant (25%) was positive for fontanel. All of them (100%) showed 0.5 - 1 degrees of fever. Three cases (75%) had vomiting. As only more than 2 years old children could express their clinical symptoms, 2 (50%) of them showed headache. Patients were negative for Kernig, Redor, Brudzinski, hepatosplenomegaly, lymphadenopathy, pharyngitis and rash. 

Statistically, significant correlation was not found between clinical symptoms and positive cases. Biochemical evaluation in positive cases revealed higher ESR in all cases (100%). CRP was positive in three cases (75%). CSF glucose was normal in all cases (mean 58.25 mg/dL). CSF protein level was normal in three cases (75%), and high in one case (means 46.5 mg/dL). The mean WBC in CSF was 466.5 and 14375 WBC in the blood. CSF cell analysis was preferred in three cases with lymphocytes and one case showed higher degree of PMN. The statistically significant correlation was not found between data obtained from biochemical analysis and positive cases.

## 5. Discussion

There are a few studies in Iran that reporting the prevalence of HSV meningitis in children. This cross-sectional study was performed on children admitted to Taleghani hospital who were suspected to meningitis. In our study, 4 cases showed HSV-1 (8.8%) with no positive HSV-2 infection result. This is higher than overall rate of HSV meningitis (4%). On the other hand, our findings are different from previous reports showing HSV-2 being the most common cause of herpes simplex virus meningitis (5). Recent study in South of Iran revealed the 20% prevalence of HSV meningitis that about 7% belonged to HSV with no discrimination between type 1 and 2 (10). 

The prevalence of neonatal herpes differs among countries. It is rarely seen in the UK but has shown high incidence in USA. Neonatal herpes can be resulted from infection with either HSV-1 or HSV-2, with the latter being associated with a poorer prognosis (11, 12). Previous seroepidemiological studies in our area, only showed seropositivity against HSV-1 (13). It has been also similar with the study in Greece (14). HSV meningitis pattern is going to be changed in different studies that are showing the diversion of prevalent HSV meningitis from HSV-2 to HSV-1. The HSV-2 is the most common cause of genital herpes in most of the countries (15), where it is responsible for approximately 85% of cases, and is involved in 70% of neonatal herpes (16, 17).

We report the detection of HSV- 4 cases (8.8%) in comparison with HSV-2 which was not detected in none of patients. However, we do not have enough data from Iran, in a previous study it was showed that the incidence of HSV-1 was four time more (40 %) than HSV-2 (10 %) in CNS infections (18). This pattern has also been shown in recent studies in Greece which determined HSV-1 instead of HSV-2 in meningitis cases (8, 14, 19). Recent studies in Europe have been showed conversion of epidemiology of HSV-2 meningitis to HSV-1. HSV-1 is more frequently associated with genital herpes than other countries in Japan (16).

 Seroepidemiological study in Japan showed that 40% of primary genital herpes results from HSV-1 infection, and is relatively common occurrence of neonatal HSV-1 infection (20). We believe this result may show different epidemiological pattern of HSV meningitis like Japan. While no clear reason has been established for this difference but there could be due to culture, lifestyle and health behavior of the people in this region. The rate of male suspected cases in this report were more than female, but in positive cases the rate of both genders was equal. As most of meningitis cases belonged to children younger than 2 years old (46 .7%), all positive cases of HSV meningitis had fever; this matter had been seen in 98-100% of cases (1, 21, 22). 

According to our results it seems that clinical symptoms are nonspecific and cannot be studied in children under 2 years old (23-25). In biochemical analysis as blood WBC level is high in infected cases, we have not observed in one child however it is not the matter to rule out meningitis (26). It seems that the CSF and blood analysis could largely suggest the presence of a viral agent in the samples, but there are some exceptions. This means we need more assays as well as fast and sensitive molecular diagnostic methods of viral meningitis. 

In conclusion, as HSV meningitis is treatable and molecular methods provided a rapid diagnosis of viruses in CSF, it has to be considered for management of meningitis patients. It may reduce the hospitalization rate and the use of unnecessary therapies and improve the health system.
